# nanoSQUID operation using kinetic rather than magnetic induction

**DOI:** 10.1038/srep28095

**Published:** 2016-06-14

**Authors:** Adam N. McCaughan, Qingyuan Zhao, Karl K. Berggren

**Affiliations:** 1 Massachusetts Institute of Technology, Dept. of EECS, 77 Massachusetts Ave, Cambridge, MA 02139, USA

## Abstract

We report on a method of nanoSQUID modulation which uses kinetic inductance rather than magnetic inductance to manip-ulate the internal fluxoid state. We produced modulation using injected current rather than an applied magnetic field. Using this injected current, we were able to observe the triangle-wave shaped modulation of the device critical current which was periodic according to the London fluxoid quantization condition. The measurement results also confirmed that the fluxoid state inside a superconducting loop can be manipulated using primarily kinetic inductance. By using primarily kinetic inductance rather than magnetic inductance, the size of the coupling inductor was reduced by a factor of 10. As a result, this approach may provide a means to reduce the size of SQUID-based superconducting electronics. Additionally, this method provides a convenient way to perform kinetic inductance characterizations of superconducting thin films.

The nanoSQUID is a nanoscale superconducting quantum interference device (SQUID) in which the weak-link elements are often Dayem bridges^1^ instead of Josephson junctions (JJs)^2^,^3^. These devices have have been fabricated from a number of materials, including aluminum^4^, niobium^5^,^6^,^7^, and lead^8^, and have demonstrated flux sensitivities as low as 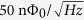
8, sufficient to resolve a single electron spin. The use of Dayem bridges instead of multilayer JJs allows the nanoSQUID to be patterned from a single-layer thin film, to reach diameters below 100 nm, and to be realized in high-*T*_c_ superconductors^9^,^10^. At these scales, the magnetic inductance of the nanoSQUID superconducting ring is small, on the order of 100 fH for a ring of size 100 nm. However, the kinetic inductance of the thin film can be significant even at these sizes, ranging from just comparable to the geometric inductance to several orders of magnitude larger.

In general, SQUID and nanoSQUID device inductances need to be controlled to implement feedback and bias. To shrink device sizes as small as possible–for applications such as superconducting electronics–it becomes convenient to use kinetic rather than geometric inductors where possible. Since kinetic inductances do not couple to magnetic fields^11^, nanoscale devices which are dominated by kinetic inductance are best controlled by injected currents. Current injection has been demonstrated before as a viable means to control SQUIDs^12^,^13^,^14^ dominated by geometric inductance. These directly-coupled SQUIDs used a large pickup loop to convert an applied magnetic field into a current bias which was injected a smaller, more sensitive readout SQUID. Although the readout SQUIDs in these devices were smaller than the pickup loops, they still used large geometric inductors to route the injected current. This injection method was also used to build a SQUID-based logic family^15^ similar to RSFQ^16^,^17^ which also used geometric inductors. Here we report the results of modulating a nanoSQUID by coupling to the device kinetic inductance instead of its magnetic inductance. We have been able to demonstrate nanoSQUID modulation without the induction of any magnetic field, by using kinetic inductance to route currents injected asymmetrically into the nanoSQUID. This approach permits similar capabilities to a field-based approach, but with reduced dimensions.

The kinetically-controlled nanoSQUID device geometry is shown in Fig. 1 and is composed of a superconducting ring, with four connecting terminals and two constrictions, all patterned on a 10-nm-thick niobium film. Terminals 1 and 4 were used to measure the switching current of the nanoSQUID, while terminals 2 and 3 were used to inject modulation current. The fabricated constrictions were 105 nm wide, ~3× larger than the coherence length of thin-film niobium, but significantly smaller than the thin-film penetration depth *λ*_thin_ = *λ*^2^/*d* where *d* is the thickness of the film and *λ* is the penetration depth^18^.

Although the constriction shown in Fig. 1 are larger than the coherence length, they still form a type of superconducting weak link: the Dayem bridge^1^. Depending on its dimensions, a Dayem bridge may have a significantly different current-phase relationship (CPR) than the typical Josephson relation *I* = *I*_c_ sin(*ϕ*). Our constrictions are wider than the coherence length, and so likely have a different CPR^19^. However, as long as the bridge cross section is significantly smaller than the thin-film magnetic penetration depth (*λ*_thin_ is ~2 μm for our 10-nm-thick Nb), the CPR of the bridge is expected to be 2*π*-periodic and allow phase slippage^20^. The exact nature of the CPR determines the method of phase slippage. In wider bridges like the ones used here, phase slippage occurs by the passage of vortices across the wire. However, the modulation technique reported here is not dependent on the form of the bridge CPR, and so should extend to narrower, more Josephson-like bridges as well.

The device was fabricated from ~10 nm niobium deposited on sapphire by DC magnetron sputtering using the process described in ref. 21. The film had a *T*_c_ of 8.2 K, a room-temperature sheet resistance of 30.4 Ω/□, and a residual resistance ratio (RRR) of 3.3. Contact pads were created by evaporating titanium and gold onto the surface using a liftoff process. The nanoSQUID geometry was then patterned by electron-beam lithography, using ~50 nm HSQ as a resist. The pattern was transferred into the film by reactive-ion etching at 50 W (distributed across a 100 mm backing wafer) for 3 min in 1.3 Pa (10 m Torr) CF_4_. All tests were performed in liquid helium at 4.2 K.

To measure the nanoSQUID characteristics, we injected a fixed modulation current *I*_mod_ into the device, as shown in the circuit schematic of Fig. 1. We then measured the switching current *I*_sw_ of the device using current applied through the bias terminals. Specifically, the *I*_sw_ discussed here represents the total amount of current passing through the constrictions just before the constrictions switched to the normal state.

The nanoSQUID *I*_sw_ distribution measurements took place with the sample submerged in a bath of liquid helium. The sample was placed in a copper-shielded sample holder. The modulation current *I*_mod_ was supplied using a variable battery source with two 20 kΩ resistors, one in series with each terminal of the battery source. With *I*_mod_ fixed, the distribution of *I*_sw_ was then measured by ramping *I*_bias_ until a nonzero voltage appeared at the *I*_bias_ terminal, indicating that the constrictions switched to the normal state. The current ramp for *I*_bias_ was provided by an arbitrary waveform generator (AWG) in series with a 10 kΩ resistor. The AWG output a 5 V_*pp*_, 200 Hz triangle wave, corresponding to a current ramp rate of ~0.3 A/s.

As we varied the injected current *I*_mod_, we observed the modulation of the device *I*_sw_ shown in Fig. 2. Since the nanoSQUID forms an unbroken superconducting loop, the shape of the *I*_sw_ modulation can be understood as follows. To maintain phase single-valuedness, current injected by *I*_mod_ splits between the two paths around the loop according to each path’s relative inductance. One of the paths has a smaller inductance, and so carries a larger fraction of *I*_mod_. The resulting imbalance of current flowing through the two constrictions reduces the total *I*_sw_ of the device. To make this analysis clearer, we can break up the contributions of *I*_mod_ into two constituent currents: *I*_+_, the portion of the modulation current which is divided equally between the two constrictions, and *I*_−_, a circulating current which has equal and opposite values through each constriction. These components are shown in Fig. 1. Since our measurement of interest, *I*_sw_, is defined as total amount of current passing through both constrictions when they switch, the measurement of *I*_+_ is automatically absorbed into *I*_sw_, leaving only *I*_−_ to affect the value of *I*_sw_. Thus, we can view the effect of *I*_mod_ as solely producing a loop current, similar to how a magnetic field would induce a loop current in a conventional SQUID. The triangle-wave pattern seen in Fig. 2 is similar to that seen in ref. 22, indicating a multi-valued, approximately-linear current-phase relationship–confirmation that the Dayem bridge constrictions are wider than the coherence length.

The periodicity of the *I*_sw_ modulation arrives from the London quantization condition, which enforces an integer number of fluxoids in the loop. When *I*_mod_ produces enough circulating current, the device can counteract the induced current by allowing a fluxoid in through one of the constrictions. Thus, the difference between adjacent maxima of the triangle wave shape correspond to *I*_mod_ inducing a circulating current equivalent to one fluxoid. One feature of note is that the distribution is not at an extrema when *I*_mod_ is zero. This distribution shift can be explained by a 4% variation in *I*_c_ between the two constrictions^23^. We additionally verified that the triangle-wave shape of the current modulation matched that of magnetic modulation by independently measuring the effect of applying a magnetic field to the device.

This device has proven to be a convenient metrological tool for extracting the kinetic inductance of superconducting thin films since it only requires low-frequency DC currents. The design of superconducting devices which have kinetic inductances often requires characterization of that inductance to achieve optimal device performance, for example tuning the L/R times of superconducting nanowire single photon detectors^24^ or nTrons^25^. Typically, these *L*_k_ values are measured by microwave reflection measurements using a network analyzer^26^, or by measuring the magnetic penetration of the film using two-coil mutual inductance measurements^27^. By patterning a kinetically-modulated nanoSQUID on the same film as these devices, it instead becomes possible to directly extract the thin-film inductance per square using only low-frequency current measurements–no microwave characterization or tunable magnetic fields are required.

Following the same principles of general SQUIDs^28^, we used the flux period to extract several parameters from the device including the total device inductance, the kinetic inductance per square, and the total inductance of each current path. To extract the material’s kinetic inductance, we assumed that the kinetic inductance per square was uniform over the entire patterned film. Kinetic inductance is expected to increase with current density^29^, but such increases are small except within a few percent of the critical current. It is likely this assumption was violated in the vicinity of the constrictions^26^, but the constrictions represent a small fraction of the total device inductance. From our numerical calculations, the inductances split *I*_mod_ such that for every 1 μA of current that flowed into the left constriction, 7.2 μA of current flowed through the right constriction, resulting in the relation *I*_−_ = 0.38 *I*_mod_. From the experimental results shown in Fig. 2 we found an 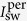 of 24.3 ± 0.1 μA, where 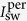 is the period of modulation of the device switching current. We then calculated the loop’s total inductance *L*_tot_ by comparing the loop current induced by 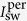 to the current that would be induced by one flux quantum, *L*_tot_/Φ_0_, and found a total inductance of 225 pH.

Since the total inductance is just the summation of the magnetic and kinetic contributions, the film’s kinetic inductance per square was then *L*_k_ = *L*_tot_ − *L*_g_, where the magnetic inductance *L*_g_ was numerically calculated, giving a value of 16.7 pH. This value was less than 10% of the total inductance, meaning if we wanted to achieve a similar inductance value with purely geometric inductance, the device loop length would need to be at least ten times larger. We then numerically calculated that there were 60.1 squares in the loop, resulting in a kinetic inductance per square of 3.7 pH/□. This sheet inductance was larger than the value predicted^26^ by 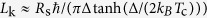 = 1.8 pH/□, where *R*_s_ is the sheet resistance just above *T*_c_ and Δ is the superconducting gap energy at 4.2 K. This difference is likely due to degradation of the film during the fabrication process, increasing *R*_s_ or decreasing the RRR.

In summary, we have demonstrated modulation of a nanoSQUID by using kinetic induction rather than magnetic induction to couple and route injected currents. By adding current asymmetrically to the two constrictions of the nanoSQUID, we were able to modulate the switching current of the device. Although the device described here has a large total inductance, and thus low sensitivity when operated as a magnetometer, this method of modulation should generalize to nanoSQUIDs of any design. This technique has applications as a means to reduce device sizes in SQUID-based supeconducting electronics.

## Additional Information

**How to cite this article**: McCaughan, A. N. *et al*. nanoSQUID operation using kinetic rather than magnetic induction. *Sci. Rep.* **6**, 28095; doi: 10.1038/srep28095 (2016).

## Figures and Tables

**Figure 1:**
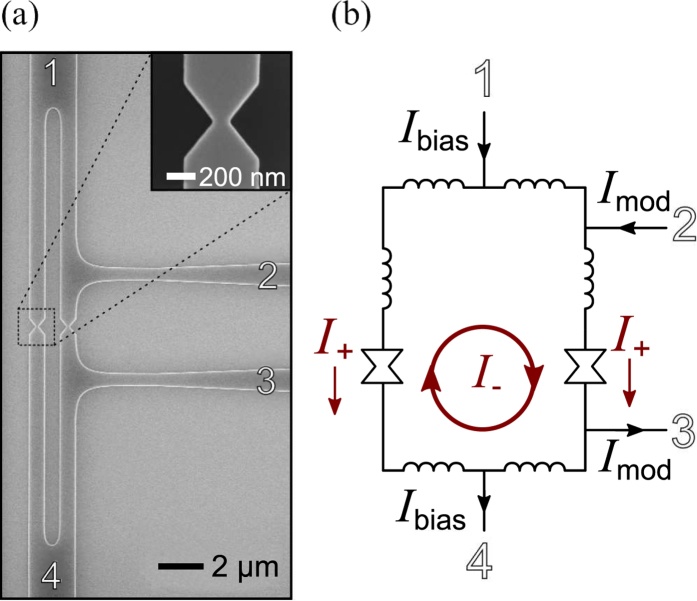
(**a**) Scanning-electron micrograph of a kinetically-controlled nanoSQUID device, fabricated from a thin niobium film. The inset shows a zoomed image one of the nanoSQUID constrictions. These constrictions were measured to be 105 nm wide at their narrowest point. (**b**) Equivalent circuit of the nanoSQUID device. Shown are the four terminals of the device and their inputs. *I*_bias_, which was used to measure the switching current of the device, flowed in from terminal 1 at the top and was carried out through terminal 4 at the bottom. The modulation current *I*_mod_ entered and left through the terminals 2 and 3 on the right. *I*_+_ and *I*_−_ are the symmetric and circulating components of *I*_mod_, respectively.

**Figure 2:**
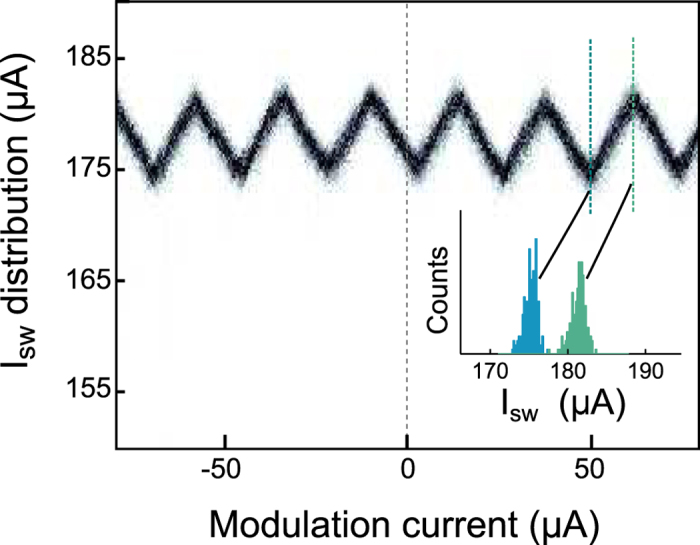
Experimental results of the nanoSQUID being modulated by injected current. Shown is the the distribution of the nanoSQUID switching current (*I*_sw_) varying as a function of the injected modulation current (*I*_mod_). Each vertical slice of the graph corresponds to a a measurement of the *I*_sw_ distribution for that value of *I*_mod_. (inset) Two slices showing the distribution of *I*_sw_ when maximally and minimally modulated by *I*_mod_.
