# Corrigendum: nanoSQUID operation using kinetic rather than magnetic induction

**DOI:** 10.1038/srep40564

**Published:** 2017-01-12

**Authors:** Adam N. McCaughan, Qingyuan Zhao, Karl K. Berggren

Scientific Reports 6: Article number: 2809510.1038/srep28095; published online: 06 14 2016; updated: 01 12 2017

The authors neglected to cite previous studies related to the use of current injection as a viable means to control SQUIDs. These additional references are listed below as references 1, 2 and 3 and should appear in the Introduction section as below.

“Current injection has been demonstrated before as a viable means to control SQUIDs^12–14^ dominated by geometric inductance. These directly-coupled SQUIDs used a large pickup loop to convert an applied magnetic field into a current bias which was injected a smaller, more sensitive readout SQUID. Although the readout SQUIDs in these devices were smaller than the pickup loops, they still used large geometric inductors to route the injected current”.

should read:

“Current injection has been demonstrated before as a viable means to control SQUIDs^1^,^2^,^3^^,12–14^ dominated either by geometric or kinetic inductances. Several of these implementations used a large pickup loop to convert an applied magnetic field into a current bias which was injected into a smaller, more sensitive readout SQUID”.

In addition, the authors would like to alert readers to a similar result published prior to final acceptance of this paper. This is listed below as reference 4.
